# Trends in Children’s Exposure to Food and Beverage Advertising on Television

**DOI:** 10.1001/jamanetworkopen.2024.29671

**Published:** 2024-08-22

**Authors:** Lisa M. Powell, Julien Leider, Rebecca M. Schermbeck, Aline Vandenbroeck, Jennifer L. Harris

**Affiliations:** 1 Division of Health Policy and Administration, School of Public Health, University of Illinois Chicago, Chicago; 2 Institute for Health Research and Policy, University of Illinois Chicago, Chicago; 3 Rudd Center for Food Policy & Health, University of Connecticut, Hartford

## Abstract

**Importance:**

Reducing children’s exposure to advertisements promoting unhealthy foods and beverages has been recognized by the World Health Organization as a key strategy to improve children’s diets and reduce childhood obesity.

**Objective:**

To examine changes in children’s exposure to food-related (food, beverage, and restaurant) television advertising, including for products high in nutrients to limit (NTL; ie, saturated fat, trans fat, total sugars, and sodium) based on federal Interagency Working Group guidelines, following changes in the voluntary industry self-regulatory Children’s Food and Beverage Advertising Initiative (CFBAI).

**Design, Setting, and Participants:**

This repeated cross-sectional study used US television ratings data on advertising exposure from The Nielsen Company for 2013, 2014, 2015, 2018, and 2022. Food and beverage product advertisements were assessed for nutritional content. Participants included 2- to 5-year-old and 6-to 11-year-old children. Data analysis was performed from July 2023 to January 2024.

**Exposure:**

Changes in CFBAI standards in 2014 and 2020.

**Main Outcomes and Measures:**

The primary outcome was the number of food-related advertisements seen per year and percentage of food and beverage product advertisements high in NTL, on all programming and children’s programming (defined as ≥35% child audience share).

**Results:**

From 2013 to 2022, total advertisements seen per year declined by 77.6% (from 4611 to 1035) for 2- to 5-year-olds and by 78.5% (from 4860 to 1046) for 6- to 11-year-olds. Advertisements seen on children’s programming decreased by 95.1% (from 1703 to 84 per year) for 2- to 5-year-olds and by 97.0% (from 1745 to 52 per year) for 6- to 11-year-olds. The percentages of advertisements for products high in NTL decreased from 2013 to 2022 but were still high: 68.9% for all programming and 63.9% for children’s programming for 2- to 5-year-olds, and 68.4% for all programming and 60.6% for children’s programming for 6- to 11-year-olds. The majority (51%-52%) of CFBAI-member food and beverage advertisements on children’s programming remained high in NTL. Black children saw more food-related television advertisements than White children (58% more for 2- to 5-year-olds and 72% more for 6- to 11-year-olds); however, the percentage of advertisements for food and beverage products high in NTL seen was similar by race.

**Conclusions and Relevance:**

In this repeated cross-sectional study of children’s exposure to food-related television advertisements, exposure via children’s programming decreased substantially. However, most advertisements seen were still for unhealthy products, and exposure from all programming remained substantial. Findings of more than 90% of advertising exposure not from children’s programming and more than 1000 food-related advertisements seen per year suggest the need for government regulations based on time of day rather than programming.

## Key Points

**Question:**

How has children’s exposure to food-related advertising on television, including for products high in nutrients to limit, changed following changes to the voluntary industry self-regulatory Children’s Food and Beverage Advertising Initiative?

**Findings:**

This repeated cross-sectional study found that, from 2013 to 2022, advertisements seen declined by 77.6% for children aged 2 to 5 years and by 78.5% for those aged 6 to 11 years, but both age groups continued to see more than 1000 advertisements per year, and the majority of food and beverage products seen were unhealthy.

**Meaning:**

These findings show that children’s exposure to food-related advertisements on television remains high, suggesting the need for government regulations based on time of day.

## Teaser

This cross-sectional study examines changes in children’s exposure to food-related television advertising after changes in the voluntary industry self-regulatory Children’s Food and Beverage Advertising Initiative standards.

## Introduction

Reducing children’s exposure to advertisements that promote unhealthy foods and beverages is a key global strategy to improve children’s diets and reduce childhood obesity. Accordingly, the World Health Organization (WHO) has called for government-mandated policies to restrict marketing of unhealthy food to children.^1^ However, few countries have enacted such policies, and industry has responded to public health concerns by introducing self-regulatory initiatives focused primarily on television advertising directed to children younger than 12 or 13 years.^2,3^ Furthermore, worldwide reviews have found limited improvements in children’s exposure to advertising of nutritionally poor products associated with food industry self-regulation.^4,5^

The US voluntary industry self-regulatory Children’s Food and Beverage Advertising Initiative (CFBAI) was introduced in 2006. Companies pledged to advertise only better-for-you products (using individual company-defined nutrition criteria) in child-directed programming.^6^ Yet following implementation, in 2009, children aged 2 to 11 years continued to view more than 4000 food-related advertisements per year.^7,8^ Moreover, nearly all (84%) food and beverage (FB) products in television advertisements viewed by children promoted products high in saturated fat, trans fat, total sugars, and/or sodium, including 98% of CFBAI-company advertisements viewed on children’s programming.^9^ Furthermore, the majority of food-related television advertisements seen by children appeared during programming that did not qualify as child-directed according to CFBAI definitions (ie, ≥35% audience share of children aged 2-11 years).^8,9,10^

In 2014, the CFBAI introduced uniform nutrition criteria with limits on calories, saturated fat, sodium and total sugars that varied by product category, with further revisions in 2020.^11^ It also updated its definition of child-directed programming to child-audience share of 30% or higher.^12^ Nonetheless, more recent evaluations found that foods and beverages advertised to children on television, including in child-directed programming, continued to be nutritionally poor.^10,13,14,15^

In addition, although previous evaluations have demonstrated reductions in children’s exposure to food-related television advertising since 2009, these reductions were largely attributed to declines in the amount of time that children spend watching traditional television (ie, commercial broadcast and cable channels).^10,14,16,17^ For example, from 2013 to 2016, the number of food-related TV advertisements viewed by children aged 2 to 5 and 6 to 11 years declined by 13% for both age groups, whereas TV viewing declined by 15% for preschoolers aged 2 to 5 years and by 20% for children aged 6 to 11 years over this time period.^10^

Evaluations have also identified limitations in CFBAI protections for more vulnerable children. Although most CFBAI companies promise not to advertise to children younger than 6 years, young children (aged 2-5 years) viewed only 5% to 7% fewer total food-related TV advertisements compared with somewhat older children (aged 6-11 years) in 2016, including on children’s television channels.^10^ CFBAI pledges also fail to recognize the disproportionate impact of higher exposure to unhealthy food advertising by children from minoritized racial or ethnic groups and of lower socioeconomic status owing to greater screen usage.^18,19,20,21^ In addition, evidence shows that Black children see disproportionately more food advertisements, even after taking into consideration differences in amount of time spent watching television.^19^ For example, Black children (aged 2-5 and 6-11 years) viewed at least 80% more food-related TV advertisements than White children in 2017, which was higher than differences in time spent watching TV (46% more and 72% more, respectively).^19^

Given changes in CFBAI program requirements, changes in television viewing patterns and ongoing racial differences in children’s exposure to advertising, it is important to continue to evaluate trends and the status of US children’s exposure to food-related television advertising. This study uses television ratings data to provide a comprehensive examination of exposure to food-related television advertising among children (aged 2-5 and 6-11 years) from 2013 to 2022.

## Methods

### Data

 This repeated cross-sectional study followed the Strengthening the Reporting of Observational Studies in Epidemiology (STROBE) reporting guideline.^22^ This study did not require institutional review board approval or informed consent because no individual participant data were used, in accordance with 45 CFR §46. Changes in exposure to food-related television advertisements following the introduction (2014) and revision (2020) of CFBAI category-specific uniform nutrition standards were assessed using television ratings data for 2013, 2014, 2015, 2018, and 2022 licensed from The Nielsen Company. Nielsen data are based on a national sample of television-equipped households in the US (average of 41 000 households). Sampling frames for housing units are obtained from Census data and used to select housing units from which households are sampled. Household-level and person-level data are weighted by Nielsen to ensure a match between the sample and known population totals using iterative proportional fitting (ie, to provide projections for all US television households). Self-reported race is collected from sampled households as part of periodic personal interviews conducted by Nielsen.

The Nielsen Company provided television program ratings data aggregated across households for advertisements shown for each product on each program and channel. Separate ratings data were obtained for children aged 2 to 5 and 6 to 11 years, including by race. The television ratings measure the percentage of children (among households with televisions) who saw a program or advertisement. The ratings data in this study covered exposure from broadcast network, cable network, and syndicated television advertising, as well as spot television advertising shown only to local broadcast markets, from all programming (regardless of audience composition) and from children’s programming (defined as programming with ≥35% child-audience [aged 2-11 years] share), excluding Spanish-language programming. Spot television advertising was not available by race. Sensitivity analyses assessed exposure from programming with different child-audience shares (30%, 25%, and 20%).

### Food Categories and Nutritional Content Classification

Food-related products were categorized into 7 categories: beverages, cereal, snacks, sweets, other foods (eg, fruits, vegetables, meats, pasta, condiments, and so forth), fast-food restaurants (ie, Nielsen’s quick-service restaurant classification), and full-service restaurants (non–quick-service restaurants). Nutritional content was assessed for the 5 FB (ie, nonrestaurant) product categories. Information on energy and nutritional content (grams of saturated fat, trans fat, and total sugars, and milligrams of sodium) for advertised FB products was determined from the following sources: manufacturer’s website, product nutrition facts panels from food labels on grocery store websites or in person, US Department of Agriculture (USDA) Food Data Central (formerly known as the USDA Nutrient Database), or the Minnesota Nutrient Data System. Across years, we were unable to obtain nutritional information on less than 9% of the FB advertising seen by children because they were either a nonspecific FB product (eg, Dairy Association or general food company) or nutritional content was not available from our aforementioned sources.

The nutritional content of each FB product was assessed according to nutrition guidelines developed by the Federal Trade Commission, Centers for Disease Control and Prevention, Food and Drug Administration, and USDA to identify FBs that should not be marketed to children (Interagency Working Group [IWG] guidelines).^23^ Advertised FB products were categorized as either an individual or a main dish and/or meal item and were assessed for nutrients to limit (NTL) based on IWG principle B for recommended limits on saturated fat, trans fat, total sugars, and sodium. Measures were generated to indicate whether each FB product was high in each and any recommended NTL. Individual items were classified as high in saturated fat if the item contained more than 1 g per reference amount customarily consumed (RACC) or more than 15% of total calories came from saturated fats. Meals and/or main dishes were considered high in saturated fat if they contained more than 1 g of saturated fat per 100 g of the item or more than 10% of total calories from saturated fats. Milk, whole eggs, and nuts were exempted from the saturated fat guidelines. All products were limited to less than 0.5 g of trans fat per RACC for individual items or labeled serving size for meals and/or main dishes. The IWG recommended that added sugars be limited to 13 g per RACC for individual items or labeled serving size for meals and/or main dishes. However, because nutrition facts panels did not list added sugars before 2020, plain or flavored milk products and yogurt were allowed an additional 12.5 g and 16 g of sugar, for a total of 25.5 g and 29 g of total sugars, respectively, to account for naturally occurring sugars in these products. In addition, fruits and 100% fruit and/or vegetable juices were exempt from the total sugars limit. Finally, a food item was considered high in sodium if it contained more than 210 mg per RACC for individual items or 450 mg per labeled serving size for meals and/or main dishes. For foods with a small RACC (≤30 g per the *Federal Register*^24,25,26,27^), recommendations refer to the amount per 50 g of food. RACCs for the general food supply were used for all products except baby or toddler products, which used RACCs for infants and children aged 1 to 3 years.

### Statistical Analysis 

Analyses included changes in children’s (aged 2-5 and 6-11 years including by race for White and Black children) annual exposure to food-related television advertisements on all and child-directed programming and by CFBAI-company membership and food, beverage and restaurant category. Analyses also examined changes in the proportion of FB product advertisements seen for products that exceeded recommended guidelines for NTL following implementation of CFBAI revised nutrition criteria. Analyses were conducted in Stata/MP statistical software version 18.0 (StataCorp) from July 2023 to January 2024.

## Results

Table 1 shows that between 2013 and 2022, children’s total exposure to food-related advertisements on all television programming decreased by 77.6%, from 4611 to 1035 advertisements per year, among 2- to 5-year-olds and by 78.5%, from 4860 to 1046 advertisements per year, among 6- to 11-year-olds. Exposure to cereal advertisements decreased the most (by 87.4% for children aged 2-5 years and by 86.8% for children aged 6-11 years), whereas exposure to fast-food restaurant advertisements decreased the least (by 63.7% for children aged 2-5 years and by 65.7% for children aged 6-11 years). Fast-food advertisements also made up the largest category (36% for children aged 2-5 years and 38% for children aged 6-11 years) of food-related advertisements seen by children in 2022. Additionally, among FB categories, sweets represented a substantial portion of exposure (24% of nonrestaurant advertisements), whereas exposure decreased the least for beverage products (by 73.5% for children aged 2-5 years and 74.5% for children aged 6-11 years). Furthermore, of beverage advertisements seen on all programming in 2022, 54.3% seen by 2- to 5-year-olds and 54.6% seen by 6- to 11-year-olds promoted sugar-sweetened beverages (data not shown).

**Table 1.** Children’s Exposure to Food-Related Advertisements by Age, CFBAI Membership, Product Category, and Programming Audience, 2013-2022^a^

Age group and type of advertisements	All audiences						≥35% Child audience					
	2013	2014	2015	2018	2022	Change, 2013-2022, %	2013	2014	2015	2018	2022	Change, 2013-2022, %
Children aged 2-5 y												
Total No. of food-related advertisements per year	4611	4531	4041	3210	1035	−77.6	1703	1702	1358	892	84	−95.1
CFBAI companies	2727	2536	2153	1511	436	−84.0	1238	1129	836	454	24	−98.0
Non-CFBAI companies	1884	1995	1888	1699	598	−68.2	465	573	522	438	59	−87.2
Total No. of food and beverage advertisements per year	2998	2879	2510	1906	552	−81.6	1228	1181	914	627	45	−96.3
Beverages	361	332	348	374	96	−73.5	91	79	68	85	2	−97.4
Cereal	755	570	443	273	95	−87.4	583	419	315	192	14	−97.7
Snacks	339	320	265	191	59	−82.5	154	142	102	26	1	−99.4
Sweets	658	649	589	485	131	−80.1	117	117	103	141	5	−96.0
Other	885	1009	865	583	171	−80.7	283	424	327	182	24	−91.6
Total No. of restaurant advertisements per year	1613	1652	1532	1304	482	−70.1	474	521	444	265	38	−91.9
Fast-food restaurants	1036	1047	1034	904	377	−63.7	248	257	237	110	8	−96.9
CFBAI companies	334	313	267	239	58	−82.8	190	172	135	85	4	−97.7
Non-CFBAI companies	702	735	767	665	319	−54.5	58	85	101	26	3	−94.1
Full-service restaurants	577	604	498	400	106	−81.7	226	264	207	155	31	−86.5
CFBAI companies	0	0	0	0	0	NA	0	0	0	0	0	NA
Non-CFBAI companies	577	604	498	399	106	−81.7	226	264	207	155	31	−86.5
Children aged 6-11 y												
Total No. of food-related advertisements per year	4860	4725	4460	3077	1046	−78.5	1745	1787	1554	941	52	−97.0
CFBAI companies	2868	2650	2462	1525	462	−83.9	1293	1199	1034	555	27	−97.9
Non-CFBAI companies	1992	2074	1998	1553	583	−70.7	452	588	520	387	25	−94.5
Total No. of food and beverage advertisements per year	3134	2972	2800	1864	564	−82.0	1271	1236	1087	698	33	−97.4
Beverages	391	366	399	355	100	−74.5	102	101	92	84	1	−99.2
Cereal	785	613	542	319	104	−86.8	604	463	406	244	15	−97.5
Snacks	376	346	302	180	61	−83.8	174	162	125	28	0	−99.9
Sweets	730	718	687	482	134	−81.6	148	163	153	169	4	−97.1
Other	852	928	871	528	165	−80.6	243	347	311	172	13	−94.7
Total No. of restaurant advertisements per year	1726	1753	1659	1213	482	−72.1	473	551	466	243	19	−96.0
Fast-food restaurants	1151	1166	1167	865	395	−65.7	278	316	286	120	7	−97.4
CFBAI companies	363	347	302	240	63	−82.6	205	196	159	95	5	−97.4
Non-CFBAI companies	788	819	865	625	332	−57.9	73	119	127	26	2	−97.5
Full-service restaurants	575	587	492	348	87	−84.9	195	235	180	123	12	−94.1
CFBAI companies	0	0	0	0	0	NA	0	0	0	0	0	NA
Non-CFBAI companies	575	587	492	348	87	−84.9	195	235	180	123	12	−94.1

Abbreviations: CFBAI, Children’s Food and Beverage Advertising Initiative; NA, not applicable.

^a^ Data are licensed from The Nielsen Company. Totals may not exactly equal subtotals as a result of rounding.

Exposure to food-related advertisements on children’s programming decreased even more compared with declines on all programming, by 95.1% (from 1703 to 84 advertisements per year) for 2- to 5-year-olds and by 97.0% (from 1745 to 52 advertisements per year) for 6- to 11-year-olds. Thus, by 2022 less than 10% of children’s food-related advertisement exposure came from children’s programming (8% for children aged 2-5 years; 5% for children aged 6-11 years), compared with just over one-third in 2013. In sensitivity analyses of alternative definitions of children’s programming (eTable 1 in Supplement 1), the proportion of advertisements viewed on children’s programming remained low, even on programming with greater than or equal to 20% child audience-share (21% of advertisements for children aged 2-5 years and 18% of advertisements for children aged 6-11 years). Hence, regardless of the definition for children’s programming, by 2022, 80% to 90% of exposure came from non–children’s programming. In addition, exposure to advertisements from CFBAI member companies on children’s programming declined at a higher rate compared with nonparticipating companies. As a result, in 2022 CFBAI companies were responsible for just 29% of advertisements viewed on children’s programming by 2- to 5-year-olds and 53% of advertisements viewed by 6- to 11-year-old children, compared with 73% for 2- to 5-year-olds and 74% for 6- to 11-year-olds in 2013.

Assessing the nutritional content of products, Table 2 shows a decrease in the proportion of FB products that were high in NTL, with greater declines for advertisements on children’s programming and for CFBAI member companies. In 2013, more than 90% of CFBAI-member products in advertisements seen on children’s programming were high in NTL, compared with 51.0% (for children aged 6-11 years) and 51.5% (for children aged 2-5 years) in 2022. However, the majority of FB product advertisements seen in 2022 on all programming and children’s programming was still for products high in NTL: 68.9% for all programming and 63.9% for children’s programming of products for 2- to 5-year-olds, and 68.4% for all programming and 60.6% for children’s programming for 6- to 11-year-olds. eTable 2 in Supplement 1 shows the greatest reductions for products high in total sugars, particularly on children’s programming, where the proportion of advertisements seen for high-in-sugar products decreased from 79.5% to 38.8% for 2- to 5-year-olds and from 82.4% to 45.0% for 6- to 11-year-olds. The Figure summarizes trends in children’s exposure to food-related advertisements.

**Table 2.** Percentage of Food and Beverage Products in Television Advertisements Seen by Children High in Nutrients to Limit, by Age, Product Category, CFBAI Membership, and Programming Audience, 2013-2022^a^

Age group and type of advertisements	All audiences					≥35% Child audience				
	2013	2014	2015	2018	2022	2013	2014	2015	2018	2022
Children aged 2-5 y										
All foods and beverages	80.4	78.6	78.0	69.9	68.9	90.2	83.0	80.7	68.4	63.9
Beverages	36.7	31.2	37.4	37.4	41.4	45.9	21.8	2.0	2.8	0.2
Cereal	86.3	86.7	74.2	47.9	38.0	97.7	98.0	84.1	54.1	31.0
Snacks	88.7	88.8	91.9	76.3	77.9	99.1	96.8	98.9	99.4	100.0
Sweets	91.1	91.6	90.0	89.8	93.3	99.6	98.7	95.9	98.4	100.0
Other	82.9	78.2	84.5	83.0	80.2	81.5	70.4	84.0	87.6	81.0
By CFBAI membership										
CFBAI companies	83.6	81.1	80.4	69.5	69.5	92.0	83.9	82.4	59.2	51.5
Non-CFBAI companies	68.3	70.0	70.5	70.6	67.8	80.7	79.4	75.1	82.0	73.7
Children aged 6-11 y										
All foods and beverages	82.3	81.3	78.6	70.3	68.4	93.8	89.2	81.7	69.5	60.6
Beverages	40.6	33.3	37.4	37.7	42.6	57.8	28.8	3.7	3.2	0.4
Cereal	87.4	88.4	76.2	50.4	37.3	98.2	98.4	84.5	55.2	29.8
Snacks	88.8	90.0	92.4	78.1	77.9	99.2	98.0	98.6	99.4	100.0
Sweets	91.2	91.7	90.1	90.5	93.2	99.3	98.2	96.7	98.2	100.0
Other	86.7	84.5	85.9	83.8	80.5	92.0	86.6	87.8	89.6	87.5
By CFBAI membership										
CFBAI companies	85.1	83.8	80.2	69.0	68.9	94.6	89.8	81.4	60.5	51.0
Non-CFBAI companies	71.3	72.5	73.2	73.1	67.3	89.8	86.3	82.8	87.1	79.3

Abbreviation: CFBAI, Children’s Food and Beverage Advertising Initiative.

^a^ Data are licensed from The Nielsen Company. It could not be determined whether certain food and beverage products were high in nutrients to limit; this affected less than 9% of food and beverage advertising seen by children aged 2 to 5 and 6 to 11 years across years. Those products are not included in the denominator of the percentages in this table. Restaurant advertising was not assessed for nutritional content and is not reflected in this table.

**Figure.**
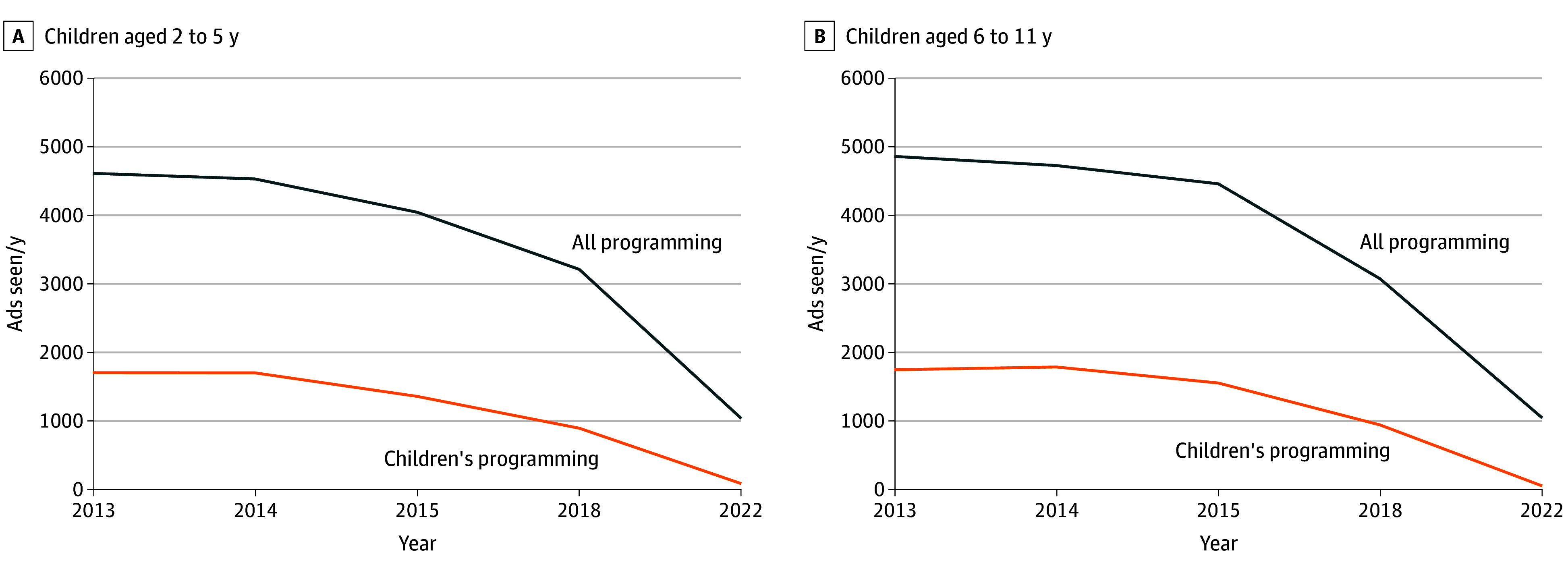
Children’s Exposure to Food-Related Advertisements, by Age and Programming Audience, 2013-2022 Data are licensed from The Nielsen Company.

Analyses by race (Table 3) showed similar declines in food-related advertisements seen by Black compared with White children from 2013 to 2022 (for children aged 2-5 years, there were decreases of 81.4% for Black children and 78.1% for White children; for children aged 6-11 years, there were decreases of 78.3% for Black children and 79.8% for White children). However, Black children in both age groups continued to view substantially more such advertisements compared with their White peers (1380 vs 876 advertisements per year for children aged 2-5 years and 1506 vs 877 advertisements per year for children aged 6-11 years). Although the disparity in advertisements viewed by Black vs White children declined for 2- to 5-year-olds (from 85% more advertisements seen in 2013 to 58% more in 2022), it increased for 6- to 11-year-olds (60% more in 2013 vs 72% more in 2022). However, the proportion of products high in NTL was similar for advertisements seen by Black and White children in all programming and children’s programming (eTable 3 in Supplement 1). There were also racial differences in time spent watching television (25% more for children aged 2-5 years and 62% more for children aged 6-11 years for Black vs White children; television viewing data from The Nielsen Company not shown in tables).

**Table 3.** Children’s Exposure to Food-Related Advertisements by Race, Age, CFBAI Membership, Product Category, and Programming Audience, 2013-2022^a^

Race, age group, and type of advertisement	All audiences						≥35% Child audience					
	2013	2014	2015	2018	2022	Change, 2013-2022, %	2013	2014	2015	2018	2022	Change, 2013-2022, %
Black children												
Children aged 2-5 y												
Total No. of food-related advertisements per year	7406	7720	6871	5052	1380	−81.4	2436	2353	2084	1307	99	−95.9
CFBAI companies	4491	4425	3875	2549	623	−86.1	1834	1596	1351	693	36	−98.0
Non-CFBAI companies	2915	3295	2997	2503	757	−74.0	602	757	733	614	63	−89.6
Total No. of food and beverage advertisements per year	4929	5029	4426	3115	770	−84.4	1795	1663	1459	927	57	−96.8
Beverages	593	591	606	609	144	−75.7	123	112	105	121	2	−98.0
Cereal	1163	907	753	427	127	−89.1	874	604	527	294	19	−97.8
Snacks	572	566	485	323	90	−84.2	238	210	167	39	1	−99.6
Sweets	1220	1305	1168	847	185	−84.9	161	168	163	217	6	−96.1
Other	1380	1659	1414	910	225	−83.7	399	569	496	256	28	−93.1
Total No. of restaurant advertisements per year	2477	2691	2445	1936	610	−75.4	641	690	625	380	42	−93.5
Fast-food restaurants	1618	1750	1679	1368	483	−70.1	355	357	351	167	13	−96.2
Full-service restaurants	859	942	766	568	127	−85.3	286	333	275	213	29	−90.0
Children aged 6-11 y												
Total No. of food-related advertisements per year	6936	7289	7259	5180	1506	−78.3	2245	2479	2246	1546	104	−95.4
CFBAI companies	4163	4219	4150	2695	704	−83.1	1670	1692	1511	914	59	−96.5
Non-CFBAI companies	2773	3070	3108	2485	802	−71.1	575	788	735	632	45	−92.1
Total No. of food and beverage advertisements per year	4576	4703	4663	3238	853	−81.4	1634	1730	1580	1140	69	−95.8
Beverages	579	583	662	616	146	−74.8	128	138	128	139	1	−99.0
Cereal	1046	907	836	527	167	−84.1	775	653	602	395	33	−95.8
Snacks	548	549	512	319	91	−83.3	228	230	184	46	0	−99.8
Sweets	1188	1265	1283	878	203	−82.9	190	229	219	277	10	−94.9
Other	1215	1399	1370	897	246	−79.8	313	481	447	282	25	−92.1
Total No. of restaurant advertisements per year	2360	2585	2596	1942	654	−72.3	611	749	666	406	35	−94.2
Fast-food restaurants	1574	1728	1828	1385	529	−66.4	364	429	406	199	17	−95.3
Full-service restaurants	786	857	768	556	125	−84.2	248	321	261	207	18	−92.7
White children												
Children aged 2-5 y												
Total No. of food-related advertisements per year	4010	3984	3555	2976	876	−78.1	1614	1660	1289	868	70	−95.7
CFBAI companies	2424	2295	1942	1439	379	−84.4	1171	1113	800	440	21	−98.2
Non-CFBAI companies	1586	1689	1613	1538	497	−68.6	443	548	490	428	49	−89.0
Total No. of food and beverage advertisements per year	2673	2594	2263	1836	482	−82.0	1167	1160	871	609	39	−96.6
Beverages	310	282	308	359	83	−73.2	86	73	64	83	2	−97.3
Cereal	702	546	416	269	87	−87.7	553	417	303	190	12	−97.8
Snacks	309	294	243	183	52	−83.2	147	142	99	24	1	−99.5
Sweets	551	550	507	452	118	−78.6	110	111	93	134	4	−95.9
Other	802	922	789	573	142	−82.3	272	417	312	177	20	−92.8
Total No. of restaurant advertisements per year	1336	1390	1292	1141	395	−70.5	447	501	418	259	31	−93.1
Fast-food restaurants	832	853	849	779	308	−63.0	229	242	217	105	6	−97.3
Full-service restaurants	504	537	444	362	86	−82.9	217	259	201	154	24	−88.8
Children aged 6-11 y												
Total No. of food-related advertisements per year	4344	4195	3967	2775	877	−79.8	1639	1702	1476	866	38	−97.7
CFBAI companies	2611	2414	2241	1406	394	−84.9	1214	1144	985	508	20	−98.4
Non-CFBAI companies	1733	1782	1726	1369	482	−72.2	425	558	491	357	18	−95.8
Total No. of food and beverage advertisements per year	2861	2704	2549	1734	483	−83.1	1193	1180	1036	639	25	−97.9
Beverages	344	315	353	330	88	−74.4	93	93	86	76	1	−99.2
Cereal	729	577	512	297	86	−88.2	567	440	389	225	11	−98.0
Snacks	351	319	280	168	54	−84.6	164	155	121	25	0	−99.9
Sweets	641	630	607	439	120	−81.3	139	157	145	155	4	−97.4
Other	795	862	796	501	135	−83.1	231	335	296	157	9	−96.1
Total No. of restaurant advertisements per year	1484	1492	1418	1041	393	−73.5	446	522	440	226	13	−97.1
Fast-food restaurants	965	967	976	737	323	−66.5	260	297	267	111	5	−98.1
Full-service restaurants	518	525	441	304	70	−86.5	185	225	173	116	8	−95.7

Abbreviation: CFBAI, Children’s Food and Beverage Advertising Initiative.

^a^ Data are licensed from The Nielsen Company. Totals may not exactly equal subtotals as a result of rounding.

## Discussion

The results of this repeated cross-sectional study show that children’s exposure to food-related advertising on television decreased substantially over the last decade, from almost 5000 advertisements per year in 2013 to approximately 1000 advertisements per year in 2022. Exposure from children’s programming decreased to an even greater extent. By 2022, more than 90% of children’s exposure to food-related television advertisements occurred on non–children’s programming compared with more than a decade ago when just under one-half of exposure came from children’s programming.^9^ Moreover, 11 years ago CFBAI-participating companies were responsible for almost 60% of children’s exposure to food-related advertisements, compared with approximately 40% in 2022.

The nutritional content of FB product advertisements seen by children on all television following the introduction of CFBAI’s category-specific uniform nutrition criteria in 2014 improved somewhat, from 8 in 10 advertisements promoting products high in saturated fat, trans fat, total sugars and/or sodium in 2013 to just under 7 in 10 in 2022. Nonetheless, more than 50% of FB product advertisements seen on children’s programming exceeded IWG NTL nutrition guidelines, including those from CFBAI-member companies. These results reflect limitations of self-regulation that have been previously identified. For example, 85% of products that CFBAI companies indicated may be advertised to children following introduction of its 2014 uniform nutrition criteria did not meet WHO guidelines for foods that should be advertised to children.^15^ Following its revised criteria in 2020, overall nutrition quality of products that CFBAI companies listed as appropriate for advertising to children did improve.^28^ However, only 35% of these listed products were actually advertised on children’s television programming, and the advertised products were statistically significantly less nutritious than listed products that companies did not advertise directly to children.^28^

The data from this study also showed that Black children continued to have higher exposure to food-related television advertisements compared with White children, although the proportion of products high in NTL was similar across these races. Although a recent report^19^ found that disproportionate exposure to food-related advertisements by Black vs White children decreased from 2017 to 2021, the data from the present study revealed that the exposure gap increased again in 2022. Furthermore, disparities in advertisement exposure in 2022 (58% more and 72% more for Black vs White children aged 2-5 years and 6-11 years, respectively) continued to exceed differences in time spent watching television (25% more and 62% more for Black vs White children; television viewing data from The Nielsen Company not shown in tables). Therefore, government restrictions of unhealthy food advertising to children would help to address diet-related health disparities due to disproportionate exposure to unhealthy food advertising among children in minoritized racial or ethnic communities.^1,29^

Also importantly, the overall reduction in exposure to food-related television advertisements was likely related to substantial reductions in children’s television viewing, which have been accompanied by increases in other screen use. From 2013 to 2020, the average time that young children (aged 0-8 years) spent watching traditional television declined by more than 50%, while their total screen media use increased by 25%.^20^ Among somewhat older children (aged 8-12 years), total screen usage increased by 21% from 2015 to 2021, but watching on a television set increased by 11% (including streaming services) from 2019 to 2021.^21^ Accordingly, children’s exposure to food-related marketing is prevalent in other media, including on digital devices. For example, viewing YouTube videos is now popular with young children younger than 9 years,^20,30,31^ and two-thirds of popular child-influencer videos feature at least 1 food-related appearance within the video content.^32^ Moreover, recent international studies^33,34^ indicate that children are frequently exposed to food marketing in digital media and social media platforms (estimates of 1560-2461 messages per year). Similar to television, most food-related marketing messages viewed online promote nutritionally poor products, including fast food, candy, sugar-sweetened beverages, and snacks.^31,32,33,34^ It will be important for future US-based work to examine children’s exposure to food-related marketing, including product nutritional content, across a variety of digital media platforms.

### Limitations

This study is subject to several limitations. First, because data obtained from The Nielsen Company were aggregated across their sample providing projections for US television households, we were unable to account for sampling variability and formally test statistical significance of observed changes. Second, we did not assess nutritional content for restaurant advertisements because these often did not highlight menu offerings with enough specificity to assess their nutritional content. Third, we did not examine additional ingredients of concern not captured in the IWG guidelines or CFBAI self-regulations including nonnutritive sweeteners (NNSs) and food dyes. Since the IWG guidelines were published, the American Academy of Pediatrics has recommended additional research on consumption of NNSs by children.^35^ For example, 52% of yogurts on the CFBAI list of products that may be advertised to children included NNSs.^13^ Additionally, some states are now seeking to limit food dyes in FB products because of their potential harm.^36,37^ Future work should monitor the extent of children’s exposure to advertising of products that include NNSs and dyes.

## Conclusions

This study found a substantial reduction in children’s exposure to food-related television advertisements yet, at the same time, demonstrated continued limitations of the CFBAI in protecting children from unhealthy television food-related advertising. First, the majority of children’s exposure continues to be for nutritionally poor products, placed by companies not participating in the CFBAI and appearing on programming not covered by CFBAI pledges (ie, primarily non–children’s programming). Furthermore, although CFBAI-member companies pledge not to advertise to children younger than 6 years, young children continue to view more than 1000 food-related advertisements per year, similar to their 6- to 11-year-old counterparts. Government regulations that restrict advertising of unhealthy foods and beverages applied to programming based on time of day rather than on child-audience share would be more effective. In addition, the finding that the nutritional content of foods and beverages that continue to be advertised, including on children’s programming by CFBAI-member companies, do not meet the IWG nutrition criteria supports the WHO recommendation for government-led criteria to limit foods and beverages marketed to children.

## Notes

Supplement 1. **eTable 1.** Total Number of Food-Related Advertisements Seen Per Year by Age and Programming Audience, 2022 **eTable 2.** Percent of Food and Beverage Products in Television Advertisements Seen by Children High in Nutrients to Limit, by Age, Product Category, and Programming Audience, 2013-2022 **eTable 3.** Percent of Food and Beverage Products in Television Advertisements Seen by Children High in Nutrients to Limit, by Race, Age, Product Category, CFBAI Membership, and Programming Audience, 2013-2022

Supplement 2.
Data Sharing Statement
