# Supplementary material for: Trends in Children’s Exposure to Food and Beverage Advertising on Television
Source: JAMA Netw Open. 2024 Aug 22;7(8):e2429671. doi: 10.1001/jamanetworkopen.2024.29671 (PMC11342137; doi:10.1001/jamanetworkopen.2024.29671)
Supplement: Supplement 1. — eTable 1. Total Number of Food-Related Advertisements Seen Per Year by Age and Programming Audience, 2022 eTable 2. Percent of Food and Beverage Products in Television Advertisements Seen by Children High in Nutrients to Limit, by Age, Product Category, and Programming Audience, 2013-2022 eTable 3. Percent of Food and Beverage Products in Television Advertisements Seen by Children High in Nutrients to Limit, by Race, Age, Product Category, CFBAI Membership, and Programming Audience, 2013-2022 [file jamanetwopen-e2429671-s001.pdf]

# Supplemental Online Content

Powell LM, Leider J, Schermbeck RM, Vandenbroeck A, Harris JL. Trends in children's exposure to food and beverage advertising on television. *JAMA Netw Open.* 2024;7(8):e2429671. doi:10.1001/jamanetworkopen.2024.29671

**eTable 1.** Total Number of Food-Related Advertisements Seen Per Year by Age and Programming Audience, 2022

**eTable 2.** Percent of Food and Beverage Products in Television Advertisements Seen by Children High in Nutrients to Limit, by Age, Product Category, and Programming Audience, 2013-2022

**eTable 3.** Percent of Food and Beverage Products in Television Advertisements Seen by Children High in Nutrients to Limit, by Race, Age, Product Category, CFBAI Membership, and Programming Audience, 2013-2022

This supplemental material has been provided by the authors to give readers additional information about their work.

© 2024 Powell LM et al. *JAMA Network Open.*

**eTable 1.** Total Number of Food-Related Advertisements Seen Per Year by Age and Programming Audience, 2022

| | All programming | ≥35% child audience | ≥30% child audience | ≥25% child audience | ≥20% child audience |
|---|---|---|---|---|---|
| Children 2-5 years of age | 1035 | 84 | 171 | 206 | 213 |
| Children 6-11 years of age | 1046 | 52 | 137 | 185 | 191 |

Data are licensed from The Nielsen Company.

**eTable 2.** Percent of Food and Beverage Products in Television Advertisements Seen by Children High in Nutrients to Limit, by Age, Product Category, and Programming Audience, 2013-2022

| | All | | | | | ≥35% child audience | | | | |
|---|---|---|---|---|---|---|---|---|---|---|
| | 2013 | 2014 | 2015 | 2018 | 2022 | 2013 | 2014 | 2015 | 2018 | 2022 |
| ***Saturated Fat*** | | | | | | | | | | |
| ***Children 2-5 years of age*** | | | | | | | | | | |
| All foods and beverages | 39.8 | 43.5 | 46.3 | 44.7 | 42.9 | 24.1 | 30.5 | 34.5 | 37.4 | 23.8 |
| Beverages | 0.5 | 0.4 | 2.4 | 2.1 | 1.2 | 0.0 | 0.0 | 1.2 | 0.2 | 0.0 |
| Cereal | 11.0 | 11.7 | 10.0 | 28.3 | 3.2 | 11.3 | 12.0 | 10.4 | 33.4 | 0.0 |
| Snacks | 44.5 | 47.3 | 54.6 | 58.4 | 73.0 | 19.8 | 19.0 | 31.5 | 47.8 | 100.0 |
| Sweets | 62.6 | 62.6 | 62.2 | 57.1 | 74.4 | 7.6 | 11.8 | 7.4 | 11.2 | 46.6 |
| Other | 61.7 | 62.6 | 70.2 | 66.1 | 54.0 | 68.0 | 66.1 | 76.4 | 79.2 | 32.6 |
| ***Children 6-11 years of age*** | | | | | | | | | | |
| All foods and beverages | 40.4 | 43.7 | 45.0 | 43.8 | 43.1 | 25.3 | 31.9 | 32.7 | 36.9 | 28.1 |
| Beverages | 0.5 | 0.3 | 2.5 | 2.1 | 1.3 | 0.0 | 0.0 | 2.0 | 0.2 | 0.0 |
| Cereal | 13.6 | 13.8 | 11.8 | 31.4 | 2.7 | 14.2 | 14.1 | 12.1 | 35.5 | 0.0 |
| Snacks | 45.2 | 46.6 | 51.8 | 57.0 | 72.8 | 22.9 | 21.0 | 28.5 | 36.8 | 100.0 |
| Sweets | 59.7 | 58.2 | 58.0 | 52.6 | 74.8 | 6.8 | 10.3 | 7.3 | 10.4 | 41.4 |
| Other | 64.7 | 68.7 | 73.6 | 68.1 | 57.5 | 76.0 | 82.8 | 84.8 | 83.9 | 58.2 |
| ***Trans Fat*** | | | | | | | | | | |
| ***Children 2-5 years of age*** | | | | | | | | | | |
| All foods and beverages | 2.5 | 2.4 | 0.8 | 0.4 | 0.9 | 1.5 | 1.6 | 0.0 | 0.0 | 0.0 |
| Beverages | 0.0 | 0.0 | 0.0 | 0.0 | 0.0 | 0.0 | 0.0 | 0.0 | 0.0 | 0.0 |
| Cereal | 0.0 | 0.0 | 0.0 | 0.0 | 0.0 | 0.0 | 0.0 | 0.0 | 0.0 | 0.0 |
| Snacks | 1.6 | 1.4 | 0.0 | 0.0 | 0.0 | 0.0 | 0.2 | 0.0 | 0.0 | 0.0 |
| Sweets | 3.8 | 3.9 | 1.0 | 1.2 | 1.0 | 8.2 | 9.1 | 0.1 | 0.1 | 0.0 |
| Other | 5.1 | 3.9 | 1.7 | 0.3 | 2.1 | 2.9 | 1.8 | 0.1 | 0.0 | 0.0 |
| ***Children 6-11 years of age*** | | | | | | | | | | |
| All foods and beverages | 2.6 | 2.5 | 0.8 | 0.4 | 0.8 | 1.6 | 1.8 | 0.1 | 0.0 | 0.0 |
| Beverages | 0.0 | 0.0 | 0.0 | 0.0 | 0.0 | 0.0 | 0.0 | 0.0 | 0.0 | 0.0 |
| Cereal | 0.0 | 0.0 | 0.0 | 0.0 | 0.0 | 0.0 | 0.0 | 0.0 | 0.0 | 0.0 |
| Snacks | 1.7 | 1.5 | 0.0 | 0.0 | 0.0 | 0.1 | 0.3 | 0.0 | 0.0 | 0.0 |
| Sweets | 3.7 | 3.8 | 0.9 | 1.1 | 1.1 | 7.5 | 7.3 | 0.0 | 0.1 | 0.0 |
| Other | 5.4 | 4.4 | 1.9 | 0.3 | 2.0 | 3.5 | 2.6 | 0.2 | 0.0 | 0.0 |
| ***Sugar*** | | | | | | | | | | |
| ***Children 2-5 years of age*** | | | | | | | | | | |
| All foods and beverages | 57.3 | 52.8 | 51.9 | 43.5 | 40.8 | 79.5 | 66.4 | 64.0 | 52.9 | 38.8 |
| Beverages | 33.9 | 28.5 | 34.2 | 34.7 | 36.0 | 45.9 | 21.8 | 2.0 | 2.6 | 0.2 |
| Cereal | 86.1 | 85.9 | 72.4 | 44.9 | 22.1 | 97.7 | 97.8 | 83.9 | 54.0 | 18.1 |
| Snacks | 28.3 | 31.9 | 41.7 | 9.4 | 7.5 | 55.3 | 65.6 | 75.7 | 2.2 | 0.0 |
| Sweets | 87.2 | 87.8 | 87.2 | 87.9 | 91.2 | 97.9 | 94.8 | 94.7 | 98.4 | 95.5 |
| Other | 29.5 | 23.8 | 25.9 | 20.9 | 26.5 | 58.7 | 33.6 | 43.6 | 46.6 | 44.9 |
| ***Children 6-11 years of age*** | | | | | | | | | | |
| All foods and beverages | 59.1 | 55.9 | 54.6 | 46.4 | 41.2 | 82.4 | 72.1 | 68.5 | 57.6 | 45.0 |
| Beverages | 38.1 | 30.8 | 34.4 | 35.2 | 37.1 | 57.8 | 28.8 | 3.7 | 3.0 | 0.4 |
| Cereal | 87.2 | 87.9 | 74.6 | 48.1 | 22.3 | 98.2 | 98.4 | 84.3 | 55.2 | 17.4 |
| Snacks | 25.2 | 31.8 | 43.8 | 9.1 | 7.4 | 47.8 | 61.0 | 75.4 | 2.3 | 0.0 |
| Sweets | 87.6 | 88.2 | 87.5 | 88.8 | 91.2 | 98.3 | 95.8 | 95.9 | 98.2 | 99.3 |

© 2024 Powell LM et al. *JAMA Network Open.*

| | | | | | | | | | | |
|---|---|---|---|---|---|---|---|---|---|---|
| Other | 31.8 | 26.4 | 27.5 | 25.0 | 26.8 | 69.0 | 42.2 | 50.3 | 56.8 | 63.7 |
| ***Sodium*** | | | | | | | | | | |
| ***Children 2-5 years of age*** | | | | | | | | | | |
| All foods and beverages | 17.7 | 18.7 | 27.3 | 22.0 | 27.2 | 10.4 | 14.5 | 32.9 | 23.0 | 37.4 |
| Beverages | 3.5 | 2.9 | 6.8 | 6.1 | 8.2 | 0.1 | 0.0 | 0.0 | 0.3 | 0.0 |
| Cereal | 0.0 | 1.1 | 47.8 | 2.5 | 24.4 | 0.0 | 0.2 | 54.7 | 0.0 | 24.4 |
| Snacks | 25.8 | 23.7 | 25.4 | 29.0 | 24.3 | 28.0 | 19.3 | 20.2 | 80.9 | 24.6 |
| Sweets | 1.6 | 0.5 | 3.1 | 2.4 | 0.7 | 0.2 | 0.0 | 0.8 | 0.0 | 0.0 |
| Other | 49.0 | 45.9 | 42.6 | 56.9 | 61.9 | 30.3 | 35.6 | 32.6 | 69.4 | 56.8 |
| ***Children 6-11 years of age*** | | | | | | | | | | |
| All foods and beverages | 18.1 | 18.7 | 28.0 | 21.7 | 26.7 | 11.7 | 15.3 | 34.5 | 22.3 | 37.0 |
| Beverages | 3.4 | 2.6 | 6.8 | 6.0 | 8.2 | 0.0 | 0.0 | 0.0 | 0.3 | 0.0 |
| Cereal | 0.0 | 0.9 | 49.6 | 1.9 | 23.8 | 0.0 | 0.2 | 55.8 | 0.0 | 22.2 |
| Snacks | 28.1 | 25.9 | 25.4 | 32.2 | 24.3 | 32.3 | 23.8 | 19.4 | 88.0 | 15.1 |
| Sweets | 1.7 | 0.5 | 3.3 | 2.3 | 0.8 | 0.3 | 0.0 | 1.2 | 0.0 | 0.0 |
| Other | 52.3 | 50.0 | 45.1 | 59.7 | 62.9 | 37.6 | 44.9 | 39.3 | 76.9 | 70.0 |

Data are licensed from The Nielsen Company. It could not be determined whether certain food and beverage products were high in nutrients to limit; this affected less than 9% of food and beverage advertising seen by children ages 2-5 and 6-11 years of age across years. Those products are not included in the denominator of the percentages in this table. Restaurant advertising was not assessed for nutritional content and is not reflected in this table.

© 2024 Powell LM et al. *JAMA Network Open.*

**eTable 3.** Percent of Food and Beverage Products in Television Advertisements Seen by Children High in Nutrients to Limit, by Race, Age, Product Category, CFBAI Membership, and Programming Audience, 2013-2022

| | All | | | | | ≥35% child audience | | | | |
|---|---|---|---|---|---|---|---|---|---|---|
| | 2013 | 2014 | 2015 | 2018 | 2022 | 2013 | 2014 | 2015 | 2018 | 2022 |
| ***Black children*** | | | | | | | | | | |
| ***Children 2-5 years of age*** | | | | | | | | | | |
| All foods and beverages | 80.9 | 79.6 | 79.0 | 71.2 | 68.3 | 91.1 | 84.8 | 81.3 | 69.0 | 60.4 |
| Beverages | 38.5 | 33.3 | 42.9 | 41.8 | 43.5 | 45.9 | 21.2 | 2.4 | 2.6 | 0.3 |
| Cereal | 85.5 | 83.9 | 73.2 | 48.4 | 34.8 | 97.7 | 97.9 | 83.3 | 54.7 | 25.5 |
| Snacks | 89.8 | 90.4 | 92.1 | 78.9 | 80.5 | 99.2 | 96.9 | 99.0 | 99.4 | 100.0 |
| Sweets | 90.0 | 90.8 | 89.1 | 88.8 | 92.5 | 99.5 | 98.2 | 96.3 | 98.3 | 100.0 |
| Other | 84.2 | 81.3 | 85.5 | 83.2 | 78.5 | 83.2 | 74.9 | 85.6 | 88.3 | 79.9 |
| By CFBAI membership | | | | | | | | | | |
| CFBAI companies | 83.6 | 81.8 | 80.8 | 70.7 | 68.6 | 92.8 | 85.6 | 82.0 | 59.6 | 46.7 |
| Non-CFBAI companies | 70.1 | 71.6 | 72.8 | 72.4 | 67.4 | 80.9 | 81.4 | 78.5 | 83.8 | 73.2 |
| ***Children 6-11 years of age*** | | | | | | | | | | |
| All foods and beverages | 82.0 | 81.5 | 79.4 | 71.3 | 67.3 | 93.7 | 89.3 | 81.9 | 69.8 | 56.4 |
| Beverages | 41.8 | 35.4 | 43.4 | 40.9 | 43.0 | 56.8 | 28.0 | 3.3 | 3.0 | 0.4 |
| Cereal | 85.8 | 86.9 | 74.9 | 50.6 | 32.6 | 98.0 | 98.3 | 84.0 | 55.8 | 23.1 |
| Snacks | 89.7 | 91.4 | 92.4 | 80.0 | 79.8 | 99.1 | 97.9 | 98.9 | 99.4 | 100.0 |
| Sweets | 90.5 | 90.8 | 89.4 | 89.7 | 93.2 | 99.1 | 97.8 | 96.9 | 98.2 | 100.0 |
| Other | 86.6 | 85.1 | 86.2 | 83.8 | 79.8 | 91.8 | 87.1 | 87.9 | 90.0 | 86.4 |
| By CFBAI membership | | | | | | | | | | |
| CFBAI companies | 84.3 | 83.7 | 80.9 | 69.9 | 67.0 | 94.4 | 89.9 | 81.6 | 60.8 | 44.6 |
| Non-CFBAI companies | 72.6 | 73.1 | 74.2 | 74.5 | 68.1 | 89.6 | 86.5 | 83.3 | 87.5 | 80.0 |
| ***White children*** | | | | | | | | | | |
| ***Children 2-5 years of age*** | | | | | | | | | | |
| All foods and beverages | 80.6 | 78.8 | 77.7 | 69.3 | 68.8 | 90.1 | 83.2 | 80.7 | 67.5 | 61.7 |
| Beverages | 36.7 | 30.9 | 35.9 | 36.5 | 42.7 | 45.3 | 21.1 | 2.2 | 2.7 | 0.2 |
| Cereal | 86.7 | 87.7 | 74.6 | 47.1 | 36.2 | 97.5 | 97.9 | 84.0 | 53.2 | 27.0 |
| Snacks | 88.8 | 88.9 | 92.2 | 75.4 | 78.7 | 99.1 | 96.7 | 99.0 | 99.4 | 100.0 |
| Sweets | 91.2 | 91.7 | 90.0 | 90.2 | 93.3 | 99.6 | 98.4 | 95.8 | 98.5 | 100.0 |
| Other | 82.7 | 77.4 | 84.1 | 82.7 | 80.3 | 81.4 | 70.5 | 83.9 | 86.5 | 80.4 |
| By CFBAI membership | | | | | | | | | | |
| CFBAI companies | 83.6 | 81.0 | 80.3 | 68.9 | 68.9 | 91.9 | 84.1 | 82.6 | 58.1 | 48.5 |
| Non-CFBAI companies | 69.2 | 70.9 | 69.7 | 70.3 | 68.6 | 80.7 | 79.3 | 74.2 | 81.6 | 72.7 |
| | All | | | | | ≥35% child audience | | | | |
| | 2013 | 2014 | 2015 | 2018 | 2022 | 2013 | 2014 | 2015 | 2018 | 2022 |
| ***Children 6-11 years of age*** | | | | | | | | | | |
| All foods and beverages | 82.4 | 81.5 | 78.5 | 70.0 | 68.8 | 93.9 | 89.1 | 81.8 | 69.3 | 59.0 |
| Beverages | 40.8 | 33.1 | 36.2 | 37.2 | 44.1 | 58.1 | 28.0 | 4.2 | 3.4 | 0.4 |
| Cereal | 87.4 | 88.5 | 76.5 | 50.1 | 36.3 | 98.1 | 98.3 | 84.5 | 55.1 | 27.1 |
| Snacks | 88.6 | 90.0 | 92.4 | 77.0 | 79.0 | 99.2 | 97.9 | 98.5 | 99.3 | 100.0 |
| Sweets | 91.1 | 91.8 | 90.0 | 90.9 | 93.1 | 99.3 | 97.7 | 96.7 | 98.3 | 100.0 |
| Other | 86.8 | 84.2 | 85.5 | 83.3 | 80.6 | 92.0 | 86.5 | 87.4 | 88.8 | 87.4 |
| By CFBAI membership | | | | | | | | | | |
| CFBAI companies | 85.1 | 83.7 | 80.2 | 68.8 | 69.1 | 94.7 | 89.9 | 81.7 | 60.1 | 48.2 |

© 2024 Powell LM et al. *JAMA Network Open.*

| | | | | | | | | | | |
|---|---|---|---|---|---|---|---|---|---|---|
| Non-CFBAI companies | 72.2 | 73.6 | 72.9 | 72.7 | 68.2 | 89.7 | 85.9 | 82.6 | 87.4 | 79.4 |

Data are licensed from The Nielsen Company. It could not be determined whether certain food and beverage products were high in nutrients to limit; this affected less than 9% of food and beverage advertising seen by children ages 2-5 and 6-11 years of age across years. Those products are not included in the denominator of the percentages in this table. Restaurant advertising was not assessed for nutritional content and is not reflected in this table. Abbreviations: CFBAI, Children's Food and Beverage Advertising Initiative.

© 2024 Powell LM et al. *JAMA Network Open.*
