# Supplementary material for: Trends in Children’s Exposure to Food and Beverage Advertising on Television
Source: JAMA Netw Open. 2024 Aug 22;7(8):e2429671. doi: 10.1001/jamanetworkopen.2024.29671 (PMC11342137; doi:10.1001/jamanetworkopen.2024.29671)
Supplement: Supplement 2. — Data Sharing Statement [file jamanetwopen-e2429671-s002.pdf]

Data Sharing Statement

Powell. Trends in Children's Exposure to Food and Beverage Advertising on Television. *JAMA Netw Open*. Published August 22, 2024. doi:10.1001/jamanetworkopen.2024.29671

Data
Data available: No

Additional Information
Explanation for why data not available: The Nielsen advertising data are not allowed to be shared per the contract with The Nielsen Company.
